# Spatial distribution and trends of anemia among pregnant women in Ethiopia: EDHS 2005–2016

**DOI:** 10.3389/fpubh.2023.1089383

**Published:** 2023-02-16

**Authors:** Molla Abate Ayele, Haile Mekonnen Fenta, Dereje Tesfaye Zike, Lijalem Melie Tesfaw

**Affiliations:** ^1^Department of Statistics, Mekidela Amba University, Mekane Selam, Ethiopia; ^2^Department of Statistics, Bahir Dar University, Bahir Dar, Ethiopia; ^3^Epidemiology and Biostatistics, School of Public Health, Faculty of Medicine, University of Queensland, Brisbane, Queensland, Australia

**Keywords:** anemia levels, ordinal logistic regression, partial proportional odds model, Ethiopia, spatial, zones

## Abstract

**Background:**

Anemia is a public health problem affecting both developed and developing nations worldwide with a significant consequence on health and economic growth. The problem is more severe in pregnant women. Hence, the main purpose of this study was to determine the factors of anemia levels among pregnant women in zones in Ethiopia.

**Methods:**

We utilized data from 2005, 2011, and 2016 Ethiopian demographic and health survey (EDHSs), a population-based cross-sectional study. The study includes 8,421 pregnant women. An ordinal logistic regression model with spatial analysis was used to explore factors of anemia levels among pregnant women.

**Result:**

About 224 (2.7%), 1,442 (17.2%), and 1,327 (15.8%) pregnant women were mild, moderate, and severely anemic, respectively. The spatial autocorrelation of anemia among the administrative zones of Ethiopia for the three consecutive was not significant. The middle wealth index of 15.9% (OR = 0.841, CI: 0.72–0.983) and richest wealth index of 51% (OR = 0.49, CI: 0.409–0.586) were less likely anemic compared to the poorest wealth index, age group of mother 30–39 was 42.9% (OR = 0.571, CI: 0.359–0.908) times less likely to be moderate and above anemic compared to <20 years, several household members 4–6 were 51% (OR = 1.51, CI: 1.175–1.94 more likely moderate and above anemic compared to 1–3.

**Conclusion:**

Over one-third of the pregnant women (34.5%) were anemic in Ethiopia. Wealth index, age group, religion, region, number of household members, source of drinking water, and EDHS were significant factors in anemia levels. The prevalence of anemia among pregnant women varied among Ethiopian administrative zones. North West Tigray, Waghimra, Oromia special woreda, West shewa, and East shewa were a high prevalence of anemia.

## Introduction

Anemia is considered a condition in which the hemoglobin (Hb) concentration falls below an established cut-off value, as evidenced by a reduced quality or quantity of red blood cells which minimizes oxygen-carrying capacity to tissue. Even the hemoglobin concentration decreases with dilution as the volume of circulating blood increases (1). According to the World Health Organization (WHO), anemia in pregnancy is defined as a Hb concentration of fewer than 11 grams per deciliter (2). Anemia during pregnancy is a major cause of morbidity and mortality in pregnant women in developed and developing countries. Although it can occur among any human population, pregnant women and young children are common victims of this hematological abnormality (3).

Anemia reduces levels of hemoglobin and favors changes in placental angiogenesis, limiting the availability of oxygen to the fetus and consequently causing potential restriction of intrauterine growth and low birth weight (4). Anemia is evaluated by measuring hemoglobin levels, rather than by clinical signs, which are less observable than for vitamin A deficiency and disorders of iodine scarcity (5). The hemoglobin deprivation due to anemia during pregnancy has serious maternal-fetal complications, which could even lead to maternal mortality (6). The main causes of anemia during pregnancy are nutritional deficiencies of iron, vitamin B12, and parasitic diseases in addition to this excessive menstrual bleeding, acute or chronic blood loss, chronic diseases, parasites infestation, hemolytic anemia, and frequent pregnancies, and also the evidence shows that anemia contributes to 20% of deaths among pregnant women (1, 7).

It is one of the most common nutritional deficiency diseases observed, globally 1.62 billion people of the world's population are anemic and about 38% accounts for pregnant women of which 46.3% of them are in Africa (8). Sub-Saharan Africa took the greatest share, where 17.2 million pregnant women were reported as anemic (9), and 41.82% were accounted for by east Africa (6). Anemia is a major and one of the greatest prevalent nutritional deficiency problems disturbing pregnant women. During pregnancy, anemia prevalence differs significantly due to the reasons of differences in socioeconomic conditions, lifestyles, and health-seeking behaviors concerning different cultures (10).

The Global data shows that 56% of pregnant women in low- and middle-income countries (LMIC) have anemia due to the absence of balanced nutrients (11). Anemia is one of the major and highly spread public health problems in developing countries including Ethiopia. It leads to different complications and difficulties for the fetus and mother during the pregnancy period. The prevalence of anemia among women decreased from 27% in 2005 to 17% in 2011 but climbed to 24% in 2016, according to the Ethiopian demography and health survey report (12). In Ethiopia, different studies were done on factors associated with anemia among pregnant women using classical models such as binary logistic regression. However, binary logistic regression accounts for the status of anemia. Besides, binary logistic regression cannot deliver sufficient information for studying the pattern of different anemia levels (12, 13). Hence, we used the ordinal logistic regression model to show the pattern of anemia levels among pregnant women. Several studies in Ethiopia studied the risk factor of anemia based on the regional level (14, 15), but this study focused on the second level of administrative area (zones) in Ethiopia.

To the best of our knowledge, some research using EDHS data on the causes of anemia in Ethiopia has been done. However, they failed to display the spatial distribution among zones and its trend over time (11–13). Therefore, this study would investigate the determinants, distribution, and trends of anemia among pregnant women in Ethiopian administrative zones based on 2005, 2011, and 2016 EDHS data.

### Operational definition

#### Hemoglobin

An iron-containing respiratory pigment of vertebrate red blood cells that consists of a globin composed of four subunits each of which is linked to a heme molecule, that functions in oxygen transport to the tissues after conversion to oxygenated form in the gills or lungs, and that assists in carbon dioxide transport back to the gills or lungs after the surrender of its oxygen.

#### Anemia

A condition in which you lack enough healthy red blood cells to carry adequate oxygen to your body's tissues.

## Methods

### Study area

The study was conducted in Ethiopia, located at 3° and 14.8° latitude, 33 and 48° longitude in the Eastern part of Africa laying between the Equator and the Tropic of Cancer (16). Ethiopia is the largest and most populated country in the Horn of Africa and the capital city is Addis Ababa (“New Flower”), located almost at the center of the country (17). Ethiopia is administratively structured into nine regional states and two city administrations. Moreover, the country has 72 administrative zones Central Statistical Agency (CSA) report, 2007.

### Source of data

This study used 2005, 2011, and 2016 EDHS data conducted in Ethiopia for the last 12 years. It was worked by the Central Statistical Agency (CSA) at the inquiry of the Federal Ministry of Health (FMoH). The EDHS is drafting every 5 years to provide health and health-related indicators at the national and regional levels in Ethiopia. The data was collected from all the nine regions and the two city administrations of Ethiopia in a representative manner. The DHS data is freely available after permission has been obtained from: https://dhsprogram.com and can be accessed following the protocols.

### Inclusion criteria

**Figure 1:**
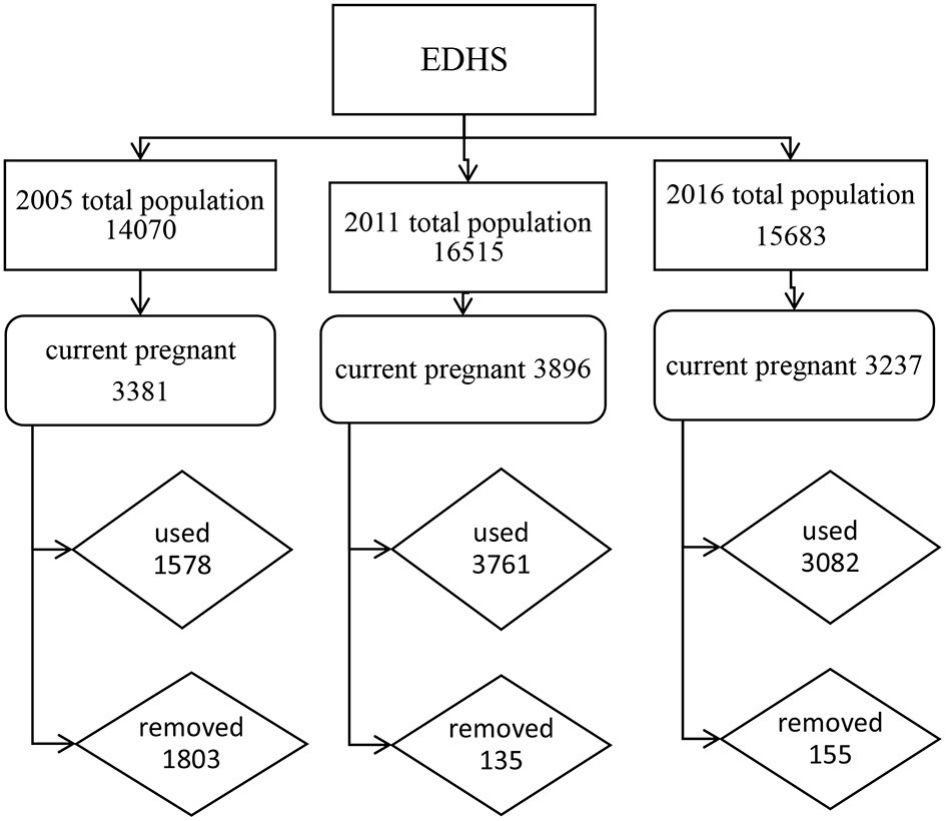
Inclusion and exclusion of pregnant women in this study.

The three surveys were conducted and anemia was included as a key indicator since the 2005 survey. In 2005, 2011, and 2016 surveys years respectively, 540 (139 urban and 401 rural areas), 624 (187 urban and 437 rural), and 645 (202 urban and 443 rural) enumeration areas (EAs) are selected using a stratified, two-stage cluster design. A total of 13,721 households (14,070 eligible reproductive age women), 16,702 households (16,515 eligible reproductive age women), and 16,650 households and 15,683 eligible reproductive-age women are selected, respectively. A total of 8,421 pregnant women of which 1,578 from 2005, 3,761 from 2011, and 3,082 from 2016 were included in this study, Figure 1.

### Exclusion criteria

All non-pregnant women whose ages are 15–49 years were excluded.

### Study variables

#### Response variable

The response variable in this study was the anemia status of pregnant women which is measured by hemoglobin level (18). The status of anemia was determined based on hemoglobin concentration in blood adjusted to the altitude. Adjusted concentration 10.0–10.9 g/dl was considered as mild anemia, 7.0–9.9 g/dl as moderate anemia, and <7.0 g/dl as severe anemia ≥11 g/dl as not anemic (6).

#### Explanatory variables

**Figure 2:**
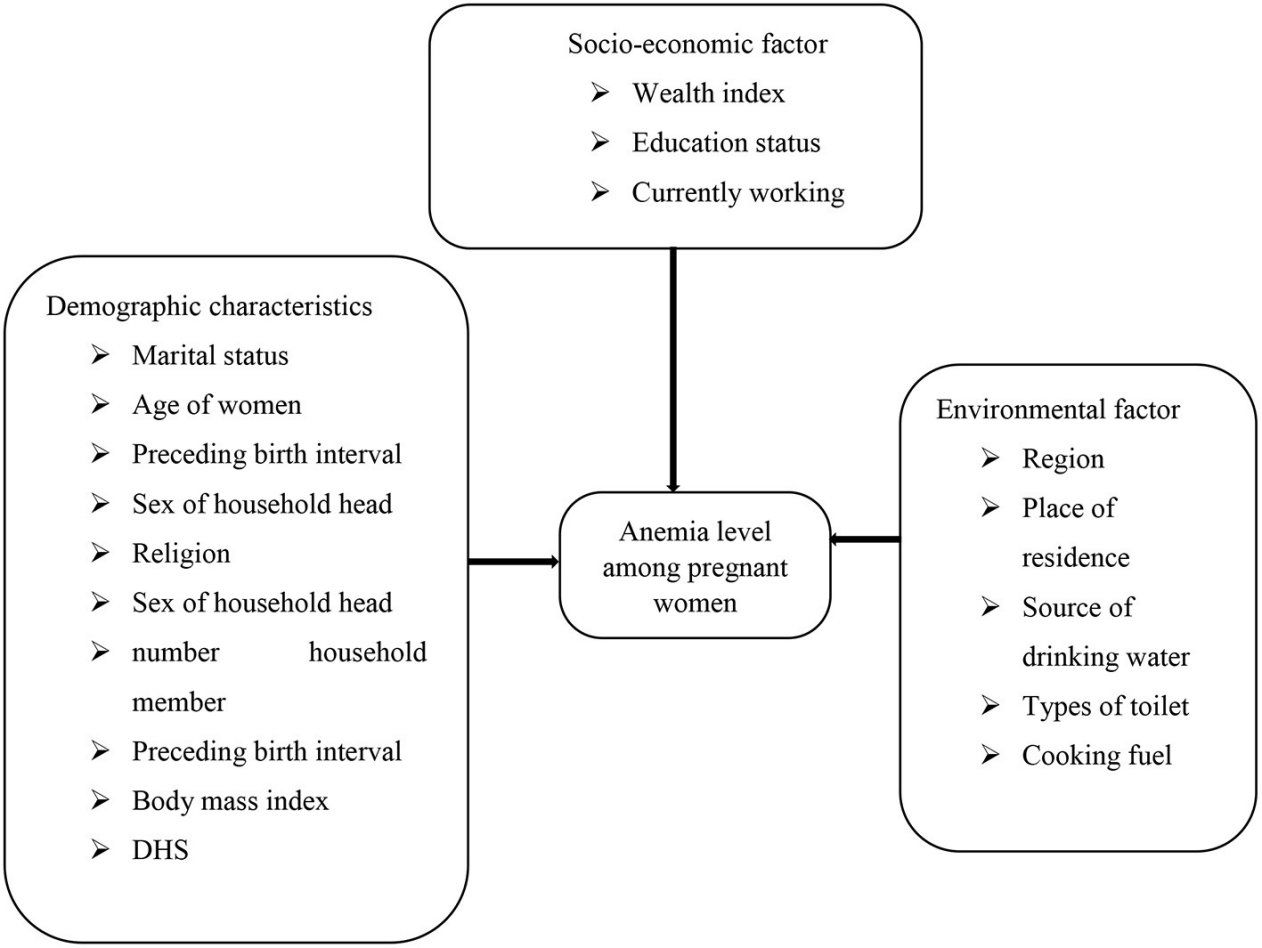
Conceptual framework for dependent and independent variables.

These variables were obtained based on insight from previous studies (1–6) (see Figure 2).

### Methods of statistical analysis

Descriptive measures were used to summarize the characteristics of the study participants using frequencies and percentages for variables.

### Ordinal logistic regression

It is applicable when a dependent variable has values with having natural order or rank (19). It is used to model the relationship between an ordinal dependent variable and a set of independent variables (20).

### Proportional odds model (POM)

It is also known as the cumulative logit model. It is used for modeling the response variable that has more than two levels with K set of explanatory variables by defining the cumulative probabilities, cumulative odds, and cumulative logits for the J-1 categories of the response, this model simultaneously uses all cumulative logits (13).

Assumed the response variable Y is a vector of an ordinal scale with J categories and **X** = (x_1_, x_2_, ..., x_p_) is the vector of covariates, then the probability of the variable response of the jth category of explanatory variable X, in particular, can be expressed by P,


(1) $$p\left[y=j/x_{1----}x_{p}\right]=\pi_{j\left(\underline{x}\right)}$$

Where Y and X are vectors.

When the response categories are ordered, the logits can utilize the ordering that results in greater power and simple interpretations. Hence, the cumulative probability of Y is the probability that falls at or below particular outcome category j is given by:


(2) $$\begin{array}{l} p\;\left(Y\leq j\right)=\;\pi_{1\;}\left(x\right)+\pi_{2\;}\left(x\right)+\ldots +\pi_{j\;}\left(x\right) \end{array}$$

j = 1, 2... J-1. Where J is categories for the response variable Y.

Then the odds of the first J −1 cumulative probability are,


(3) $$\begin{array}{l} odds\left(p\left(y\geq j\right)\right)=\left(\frac{p\left(y\geq j\right)}{1-p\left(y\geq j\right)}\right)=\frac{\pi_{j}}{1-\pi_{j}} \end{array}$$

Where *j* = 1, 2, …, *J*− 1.

The cumulative logit model (21).


(4) $$\begin{array}{l} logit\left(y_{i}\geq j/x\right)=\log\left(\frac{pr\left(y_{i}\geq j/x\right)}{pr\left(y_{i}<j/x\right)}\right)\\ \log\left(\frac{\pi_{j+1}+\pi_{j+2}+\ldots +\pi_{J}\;}{\pi_{1}+\pi_{2}+\ldots +\pi_{j}}\right)\\ =\beta_{0j}+X^{{}^{\prime }}\beta ,j=1,\ldots ,J-1 \end{array}$$

Where **β**_0*j*_ = is a threshold value.

x = set of factors or predictors.

Each cumulative logit uses all J response categories.

Manually the probability *p* (*Y* ≥ *j*), can be estimated as:


(5) $$\begin{array}{l} p\;\left(Y\;\geq j\right)=\frac{\exp^{\left(\beta_{0j}+\;x^{\prime }\;\beta \;\right)}}{1+\exp^{\left(\beta_{0j}+\;x^{\prime }\;\beta \;\right)}} \end{array}$$

The cumulative probabilities do not use the final one, P (Y ≤ j), since it must equal 1. The parameter β is a vector of regression coefficient describing the effect of corresponding covariates X on the log odds of response in category j or below. When this model good fit, it requires a single parameter rather than J-1 parameters to describe the effect of X. Because the model assumes that the effect of X is identical, proportional odds, for all J-1 cumulative logits (22).

For the ordinal regression model to hold, the assumption of parallel lines of all levels of the categorical data is satisfied since the model does not assume normality and constant variance (23).

To fit an ordinal logistic regression using the proportional odds model the assumption is that the relationship between independent variables and the dependent variable does not change for the dependent variable's categories (13).

### Partial proportional odds model

It is rare for all the explanatory variables included in the model to display the proportional odds property. A partial proportional odds model can be used when the parallel lines assumption holds or not. The partial proportional odds model bears the same characteristics as the proportional odds model but now the coefficients are associated with each category of the response variable (24). This model allows some predictors to be modeled with the POM assumption, but for those variables in which this assumption is not satisfied is with PPOM.


(6) $$\begin{array}{l} logit\left(\gamma_{j}\right)=log\left(\frac{\gamma_{j}}{1-\gamma_{j}}\right)=\eta_{i}=\theta_{j}+X_{i}^{{}^{\prime }}\beta +\tau \psi_{j} \end{array}$$

Where X is a vector containing the full set of independent variables and τ is a vector of a subset of independent variables not violating and violating parallel line assumption; β and ψ are the regression coefficients of those predictors, respectively.

### Generalized ordered model

The generalized ordered logit model is an ordinal logistic regression that considers the order of category of the response variable with a k set of explanatory variables. This model results in J-1 logits without constraining the effect of each explanatory variable is equal across the logit (25).

The model can be expressed as:


$$\begin{array}{l} \log\left(pr\left(Y>j/X\right)\right)=log\left(\frac{pr\left(Y>j/X\right)}{pr\left(Y\leq j/X\right)}\right)\\ =\alpha_{j}+\beta_{1j}x_{1}+\beta_{2j}x_{2}+\ldots +\beta_{kj}x_{k} \end{array}$$

In this model defines J – 1 sets of model parameters, one for each of the J – 1 generalized logit.

This means the model has a separate intercept (α_*j*_) and a separate set of regression parameters (β_*j*_).

This model estimates the odds of being beyond a certain category relative to being at/below that category. A positive β-value indicates that an individual is less likely to be low in the category as compared to beyond the category of the outcome variable. The generalized ordered logit model estimates the regression parameter for each independent variable on j−1 logit of the probability being at or below jth category in every logit to have different estimated values.

### Spatial analysis

Spatial analysis is an analysis that includes the influence of space in the analysis (26). It is a statistical method that is useful to identify geographical areas with the highest prevalence of anemia among pregnant women and its variability over the Ethiopian administrative zones. Ignoring such information during analysis may offer faulty results and conclusions (27).

### Spatial autocorrelation

The idea of spatial autocorrelation was proposed by Tobler in the first geography law, “Everything is related to everything else, however nearest things are related than distant things (28).” A Moran's I was used to measure spatial autocorrelation (29). The value of Global Moran's I is range from −1 to 1. When the index was distributed around – 1, the overall spatial distribution displayed is not similar and the reverse is true when the index is 1 (26, 30).

A statistically significant Moran's I (*p* < 0.05) leads to rejecting the null hypothesis (anemia among pregnant women randomly distributed) and indicates the existence of spatial autocorrelation.

The global Moran's I expressed as follows:


(8) $$\begin{array}{l} I=n\frac{\sum_{i=1}^{n}\sum_{j=1}^{n}w_{ij}\left(x_{i}-\bar{x}\right)\left(x_{j}-\bar{x}\right)}{\sum_{i=1}^{n}{w_{ij}\left(x_{i}-\bar{x}\right)}^{2}} \end{array}$$

Where *n* is the number of observations in the whole cluster, *x*_i_ and *x*_j_ are the observations at locations of i and j, $\bar{x}$ is the mean of x, and W_ij_, a component of spatial weights matrix W, is the spatial weight between locations of i and j. The Local Moran's I expressed as follows:


(9) $$\begin{array}{l} I=n\frac{\sum_{i=1}^{n}\sum_{j=1}^{n}w_{ij}\left(x_{i}-\bar{x}\right)\left(x_{j}-\bar{x}\right)}{\sum_{i=1}^{n}{\left(x_{i}-\bar{x}\right)}^{2}} \end{array}$$

### Contiguity matrix

A contiguity matrix is a matrix that explains the relationship between zones, giving the value 1 if the area i neighbor with area j, while a value of 0 is given if area i is not adjacent to the area j (31). In our study, it is referred to as matrix **W** containing w_ij_ for row i and column j. based on the contiguity of the area units. It is a square symmetric n × n matrix with (i, j) and the diagonal elements of the spatial weight matrix are zeros (32). The most common ways to construct such a matrix are as follows.


$$\begin{array}{l} \;w_{ij}=\{\begin{array}{c} 1,if\;area\;i\;and\;j\;are\;neigbhoring\\ 0,Otherwise\; \end{array}\\ \;W=\left[\begin{array}{ccccc} 0 & w_{12} & w_{13} & \cdots & w_{1N}\\ w_{21} & 0 & w_{23} & \ldots & w_{2N}\\ w_{31} & w_{32} & 0 & \ldots & w_{3N}\\ \vdots & \vdots & \vdots & \ddots & \vdots \\ w_{N1} & w_{N2} & w_{N3} & \cdots & 0 \end{array}\right] \end{array}$$

### Spatial interpolation

Kriging is the most commonly used geostatistical approach for spatial interpolation (33). It is relied on a spatial model between observations to predict attribute values at unsampled locations. One of the specificities of kriging methods is that they do not only consider the distance between observations but they also intend to catch the spatial structure in the data by comparing observations separated by specific spatial distances two at a time. In this study, the Ordinary Kriging geostatistical interpolation method was used to predict the prevalence of anemia in unobserved areas of Ethiopian administrative zones.

### Spatial ordinal logistic regression

Spatial Ordinal Logistic Regression is an analysis which is adding of spatial effects into the ordinal logistic regression model. Uses the spatial ordinal logistic regression model of the form (34):


(10) $$\begin{array}{l} logit\left(P\left(Y\leq j|X\right)\right)=\log\left[\frac{P\left(Y\leq j|X\right)}{1-P\left(Y\leq j|X\right)}\right]=\alpha_{j}+X\beta \\ +\rho W_{y} \end{array}$$

Where ρ*W*_*y*_ $=\overset{k}{\underset{j=1}{\sum }}w_{ij\;}\hat{y_{i}}/\overset{ki}{\underset{j=1}{\sum }}w_{ij}$.

Where ρ*W*_*y*_ is a form of auto-covariance and is a weighted average of the number of events among the ki neighbors. The weighting of the average location of the ijth is through W_ij_ = 1/h_ij_ where h_ij_ is the Euclidean distance between the village i and j. $\hat{y_{i}}\;is$ the alleged existence of an event and β is the regression coefficient.

The data were analyzed on SAS version 9.4 with Proc logistic command; ArcGIS version 10.4, and SPSS version 26 were used in the data management.

## Result

### Explanatory analysis

**Figure 3:**
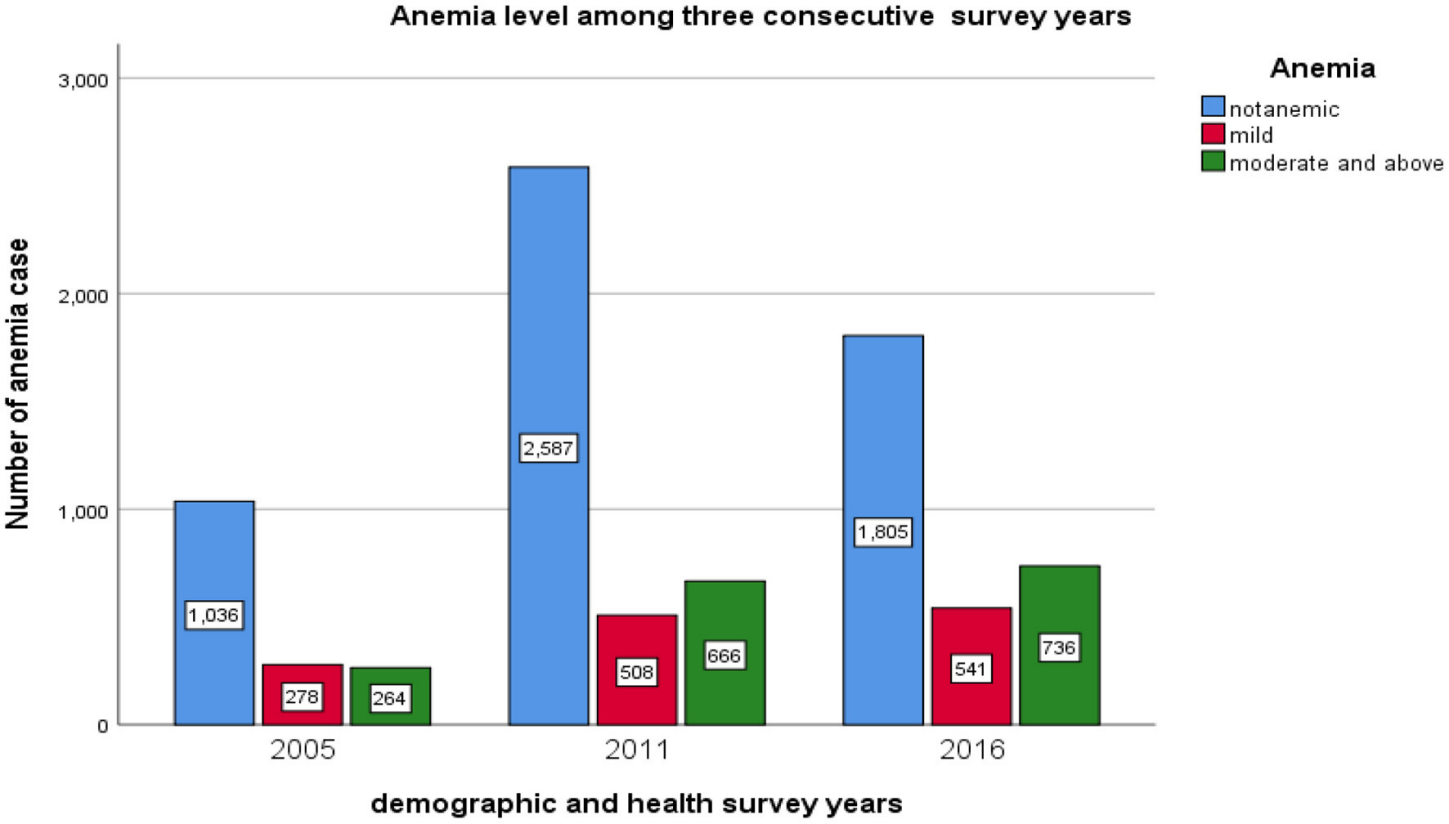
The bar chart presentation of anemia levels among pregnant women for the three consecutive EDHS data.

Among a total sample of 8,421 pregnant women considered 224 (2.7%) were severe, 1,442 (17.2%) moderate, anemic 1,327 (15.8%) mild anemic, and while among all pregnant women 5,428 (64.5%) were non-anemic, see Figure 3.

**Figure 4:**
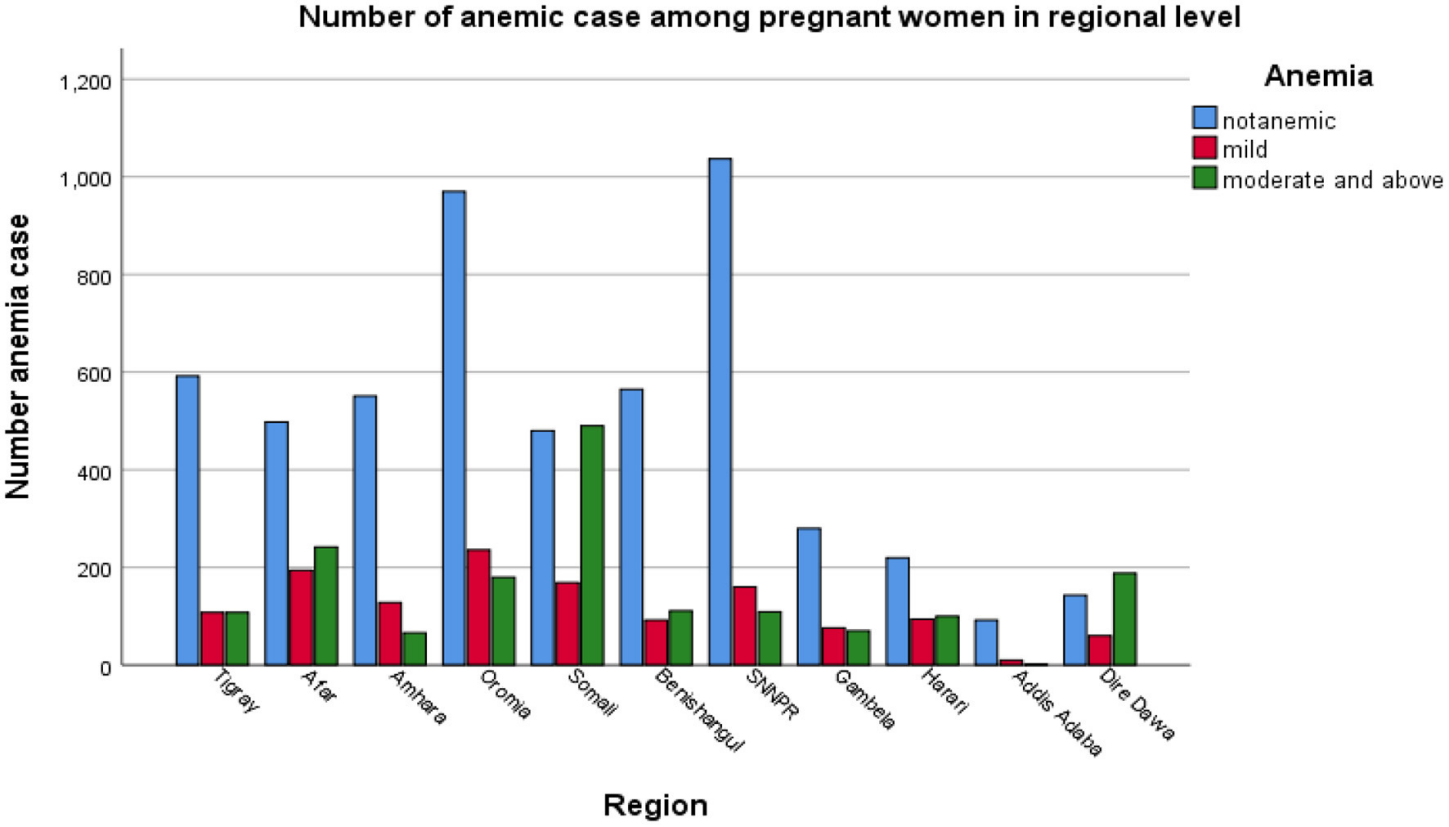
The bar chart presentation of anemia levels among pregnant women in Ethiopia's administrative regions.

The prevalence of pregnant anemic women in the survey year 2005, 2011, and 2016 were 542 (34.35%), 1,277 (33.9%), and 1,174 (38.09%), respectively (see Table 1). Besides, Figure 4 revealed the prevalence of anemic pregnant women across the region. The highest number of anemic women was noticed in Somali, Afar, and Dire Dawa.

**Table 1:** The prevalence of anemia among pregnant women in 2005, 2011, and 2016 EDHS data.

**Year**	**Frequency (%)**
2005	542 (34.35%)
2011	1,277 (33.9%)
2016	1,174 (38.09%)

About 4,290 (50.94%) were found to be aged 30–39 years. The majority of 6,277 (74.54%) mothers did not have formal education. Among the total, 6,394 (76%) of the pregnant women were not working, and 3,920 (46.55%) were Muslim. Out of the total, 67.24% used unimproved water sources. The majority of the pregnant women (90.25%) lived in rural areas, and 2,783 (33.04%) had the poorest wealth index. Concerning the number of household members, having four up to six-member households covers 4,027 (47.82%) of the total and more than half of the pregnant women used wood/straw cooking fuel (Table 2).

**Table 2:** Frequency distribution of independent variables among the 3 survey years.

**Variables**	**Categories**	**2005** **Frequency (%)**	**2011** **Frequency (%)**	**2016** **Frequency (%)**
Residence	Urban	44 (2.8)	273 (7.6)	359 (11.6)
	Rural	1,534 (97.2)	3,343 (92.4)	2,723 (88.4)
Education	No education	1,320 (83.7)	2,644 (73.2)	2,313 (75)
	Primary	231 (14.6)	928 (25.7)	616 (20)
	Secondary	49 (1.2)	25 (0.7)	106 (3.5)
	Higher	8 (0.5)	16 (0.5)	47 (1.5)
Religion	Orthodox	554 (35.1)	1,102 (30.5)	680 (22.1)
	Catholic	10 (0.6)	84 (2.3)	13 (0.4)
	Protestant	320 (20.3)	919 (25.4)	479 (15.5)
	Muslim	638 (40.4)	1,446 (40)	1,836 (59.6)
	Traditional	37 (2.3)	33 (0.9)	55 (1.8)
	Other	19 (1.2)	33 ()0.9	19 (0.6)
Wealth	Poorest	498 (31.6)	938 (25.9)	1,347 (43.7)
	Poorer	334 (21.2)	819 (22.6)	534 (17.3)
	Middle	314 (19.9)	697 (19.3)	423 (13.7)
	Richer	258 (16.3)	735 (20.3)	423 (13.7)
	Richest	174 (11)	428 (11.8)	355 (11.5)
Region	Tigray	214 (13.6)	249 (6.9)	196 (6.4)
	Afar	70 (4.4)	49 (1.4)	358 (11.6)
	Amhara	158 (10)	576 (15.9)	280 (9.1)
	Oromiya	387 (24.5)	1,590 (44)	459 (14.9)
	Somali	111 (7)	155 (4.3)	640 (20.8)
	Benishangul-gumz	128 (8.1)	55 (1.5)	224 (7.3)
	SNNP	318 (20.2)	886 (24.5)	413 (13.4)
	Gambella	71 (4.5)	8 (0.2)	177 (5.7)
	Harari	87 (5.5)	7 (0.2)	169 (5.5)
	Addis Ababa	17 (1.1)	28 (0.8)	42 (1.4)
	Dire-Dawa	17 (1.1)	13 (0.4)	124 (4)
Working	Yes	321 (20.3)	976 (26%)	730 (23.7)
	No	1,257 (79.7)	2,785 (74%)	2,352 (76.3)
Age category mother	<20	18 (1.1)	52 (1.4)	31 (1)
	20–29	515 (32.6)	1,349 (35.8)	1,202 (39)
	30–39	845 (53.5)	1,861 (49.5)	1,584 (51.4)
	40–49	200 (2.7)	500 (13.3)	265 (8.6)
Nhmember	1–3	114 (7.2)	304 (8.1)	287 (9.3)
	4–6	734 (46.5)	1,888 (50.2)	1,405 (45.6)
	7–9	594 (37.6)	1,264 (33.6)	1,142 (37.1)
	≥10	136 (8.6)	305 (6.1)	246 (8)
Preceding birth interval	≤ 24	432 (27.4)	1,078 (28.7)	1,005 (32.6)
	≥25	1,146 (72.6)	2,683 (71.3)	2,077 (67.4)
Source of drinking water	Improved	782 (49.6)	1,729 (46)	1,248 (40.5)
	Unimproved	796 (50.4)	2,032 (54)	2,834 (59.5)
Body mass index	Under weight	106 (6.7)	368 (10.2)	423 (13.7)
	Normal	1,409 (89.3)	2,947 (81.5)	2,251 (73.1)
	Overweight	63 (4)	301 (8.3)	408 (13.2)
Toilet	Improved	110 (7)	537 (14.3)	412 (13.4)
	Unimproved	1,468 (93)	3,224 (85.7)	2,670 (86.6)
Cooking fuel	Wood/straw	36 (2.3)	3,318 (88.2)	2,715 (88.1)
	Other	1,542 (97.2)	443 (11.8)	367 (11.9)

The chi-square statistics presented in Table 3 indicate anemia level among pregnant women was significantly associated with categorical predictor variables such variables are highly associated with anemia levels among pregnant women. The region, wealth index, number of household members, age category, religion, pregnant currently working, education level of the mother, cooking fuel, survey year, source of drinking water, types of toilet, body mass index, and preceding birth interval (*p*-values < 0.05).

**Table 3:** The association of socio-demographic, socio-economic, and environmental variables with anemia level among pregnant women.

		**Not anemic**	**Mild**	**Moderate and above**	***X*** **^2^ and *p*-value**
**Predictor**	**Category**	**Frequency (%)**	**Frequency (%)**	**Frequency (%)**	
Place of residence	Urban	547 (66.5)	109 (13.2)	167 (20.3)	4.347 (0.114)
	Rural	4,881 (64.2)	1,218 (16)	1,499 (19.7)	
Education	No education	4,088 (62.5)	1,070 (16.4)	1,380 (21.1)	56.928 (<0.001)
	Primary	1,163 (70.9)	219 (13.3)	259 (15.8)	
	Secondary	122 (71.3)	32 (18.7)	17 (9.9)	
	Higher	55 (77.5)	6 (8.5)	10 (14)	
Religion	Orthodox	1,580 (74.2)	307 (14.4)	245 (11.5)	407.205 (<0.001)
	Catholic	70 (89.7)	4 (5.1)	4 (5.1)	
	Muslim	2,503 (55.7)	778 (17.3)	1,214 (27)	
	Protestant	1,121 (73.8)	218 (14.4)	180 (11.8)	
	Traditional	84 (70)	20 (16.7)	16 (13.3)	
	Other	70 (90.9)	0	7 (9.1)	
Wealth index	Poorest	1,888 (58)	537 (16.5)	828 (25.5)	150.22 (<0.001)
	Poorer	984 (64.1)	260 (16.9)	292 (19)	
	Middle	899 (70.3)	187 (14.6)	192 (15)	
	Richer	891 (68.4)	216 (16.6)	196 (15)	
	Richest	766 (72.9)	127 (12.1)	158 (15)	
Region	Tigray	592 (73.3)	108 (13.4)	108 (13.4)	008.533 (< 0.001)
	Afar	498 (53.3)	(194 (20.8)	242 (25.9)	
	Amhara	551 (74)	128 (17.2)	66 (8.9)	
	Oromiya	970 (70)	236 (17)	180 (13)	
	Somali	480 (42.1)	169 (14.8)	490 (43)	
	Ben-gumz	565 (73.6)	92 (12)	111 (14.5)	
	SNNP	1,037 (79.4)	160 (12.3)	109 (8.3)	
	Gambella	280 (65.7)	76 (17.8)	70 (16.4)	
	Harari	220 (53.1)	94 (22.7)	100 (24.2)	
	Addis Ababa	92 (88.5)	10 (9.6)	2 (1.9)	
	Dire Dawa	143 (36.6)	60 (15.3)	188 (48.1)	
Pregnant currently working	Yes	1,370 (67.6)	311 (15.3)	346 (17.1)	14.29 (<0.001)
	No	4,058 (63.5)	1,016 (15.9)	1,320 (20.6)	
Age group of mother	<20	62 (61.4)	12 (11.9)	27 (26.7)	24.022 (<0.001)
	20–29	1,994 (65.0)	455 (14.8)	616 (20.1)	
	30–39	2,699 (62.9)	718 (16.7)	873 (20.3)	
	40–49	673 (69.7)	142 (14.7)	150 (15.5)	
Number of household member	1–3	456 (64.7)	124 (17.6)	125 (17.7)	29.863 (<0.001)
	4–6	2,560 (63.6)	674 (16.7)	793 (19.7)	
	7–9	1,964 (65.5)	459 (15.3)	577 (19.2)	
	≥10	448 (65)	70 (10.2)	171 (24.8)	
Preceding birth interval	≤ 24	1,546 (61.5)	383 (15.2)	586 (23.3)	28 (<0.001)
	≥25	3,882 (65.7)	944 (16)	1,080 (18.3	
Body mass index	Underweight	653 (66)	150 (15.2)	186 (18.8)	12.589 (0.013)
	Normal	4,280 (64.8)	1,045 (15.8)	1,280 (19.4)	
	Overweight	495 (59.9)	132 (15.9)	200 (24.2)	
Toilet	Unimproved	4,784 (65.1)	1,153 (15.7)	1,407 (19.2)	13.452 (<0.001)
	Improved	635 (60)	174 (16.4)	250 (23.6)	
Source of drinking water	Improved	2,440 (64.9)	629 (16.7)	690 (18.4)	11.31 (0.004)
	unimproved	2,988 (64.1)	698 (15)	976 (20.9)	
Cooking fuel	Other	1,509 (64.2)	404 (17.2)	439 (18.7)	6.279 (0.043)
	Wood/straw	3,919 (64.2)	923 (15.2)	1,227 (20.2)	

### Spatial analysis

#### Spatial distribution of anemia

**Figure 5:**
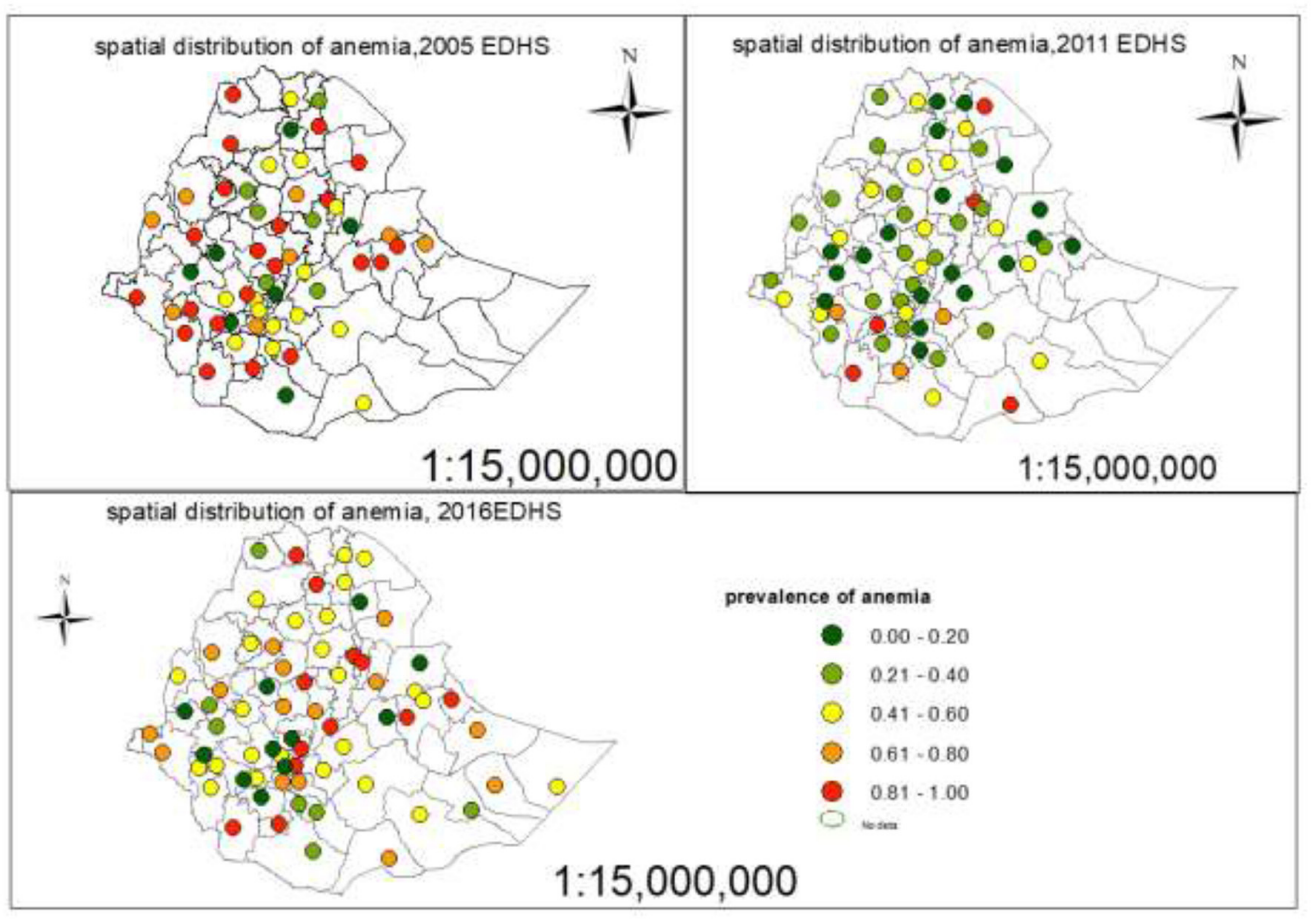
Spatial distribution of anemia on pregnant women in Ethiopian administrative zones.

Each point on the map was characterized by the prevalence of anemia in each zone. The leaf green color indicates the zones with a low distribution of anemia, whereas the mars red color indicates zones with a high distribution of anemia (Figure 5).

#### Spatial interpolation of anemia

**Figure 6:**
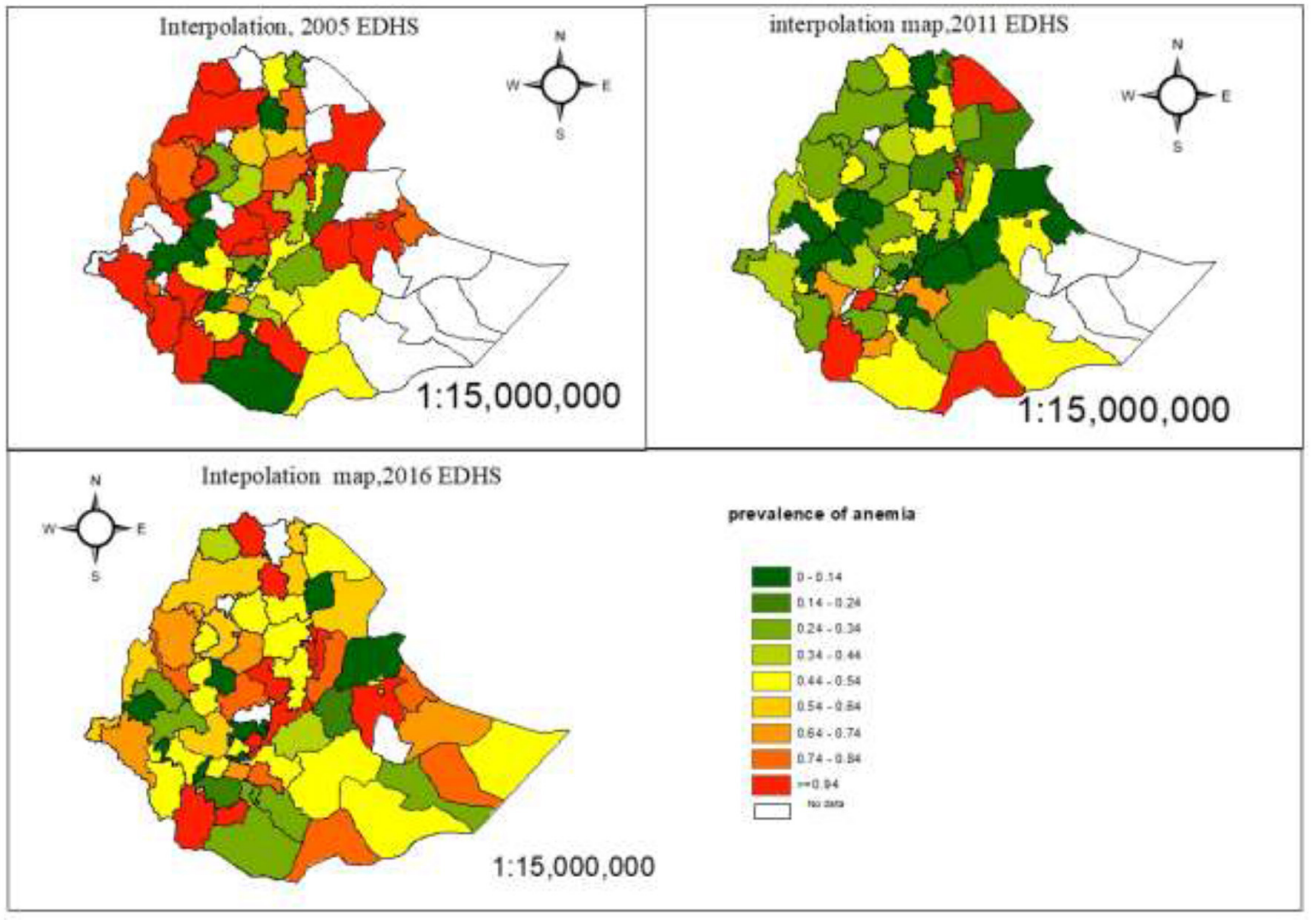
The estimated prevalence of anemia among pregnant women in Ethiopian administrative zones.

Ordinary Kriging interpolation technique, the mars red ramp color on the map indicates the predicted highest prevalence of anemia and the leaf green ramp color on the map indicates the lowest predicted prevalence of anemia (see Figure 6). Based on the result of predicted values for the prevalence of anemia in 2005, the southern part of Ethiopia would be affected by anemia. The prevalence of anemia has decreased since the 2011 EDHS, but the predicted prevalence of anemia in 2016 shows that the distribution of anemia has increased in Ethiopian administrative zones.

### Hot spot analysis

**Figure 7:**
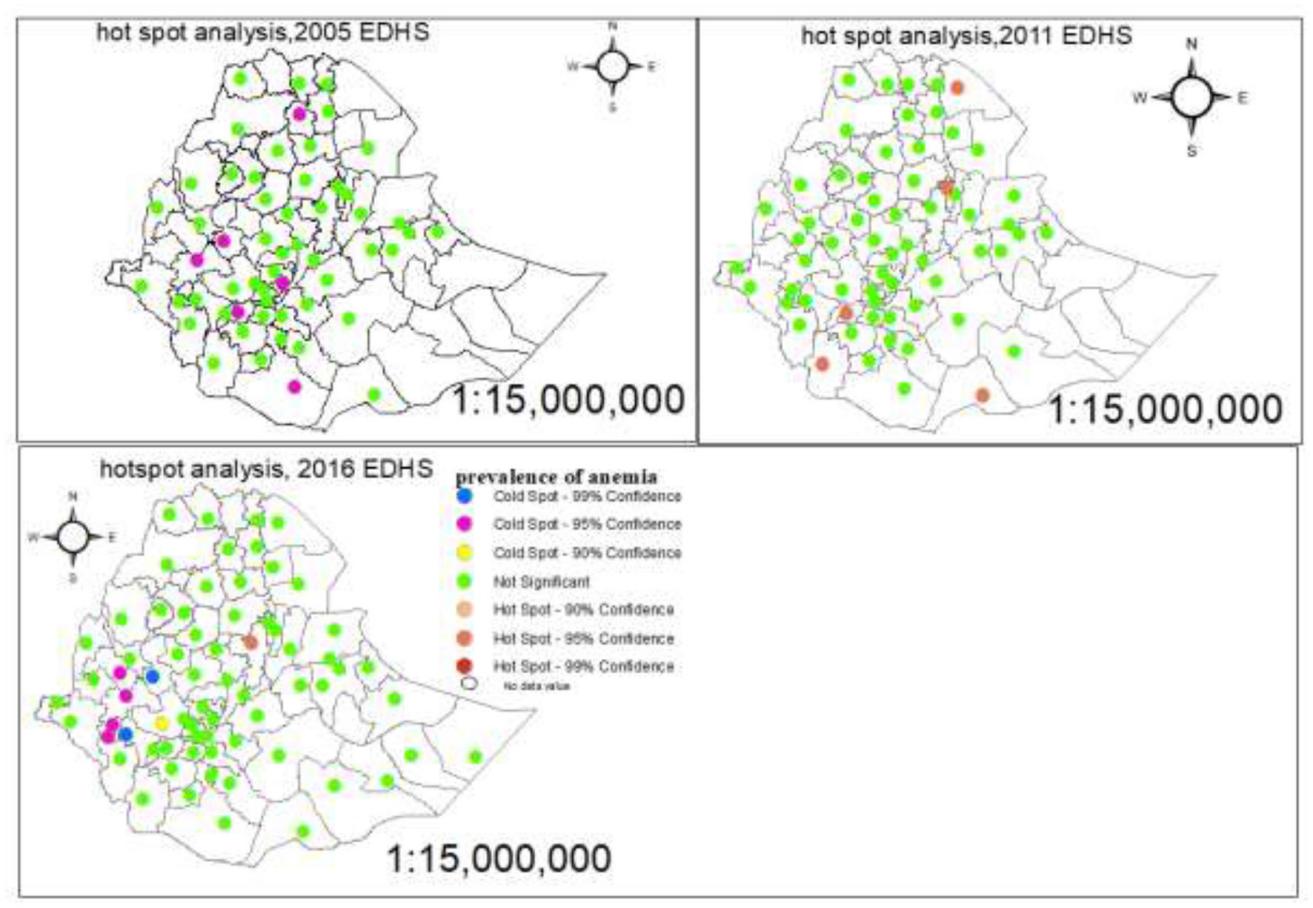
Hot spot and cold spot identification of anemia among pregnant women in Ethiopia administrative zones.

A hot spot analysis was performed to identify the high-risk and low-risk areas for the prevalence of anemia among pregnant women in Ethiopian administrative zones. The red color indicates significant hot spot (high-risk) areas for anemia and the blue color indicates the cold spot (low-risk) areas of anemia (Figure 7).

### Spatial autocorrelation, Moran's tests

We can test the spatial autocorrelation by using global moans and variogram tests. The Global Moran's test revealed that there is no spatial autocorrelation of anemia among the administrative zones of Ethiopia for the three consecutive EDHSs data sources (Table 4).

**Table 4:** Variogram output of autocorrelation statistics across 2005, 2011, and 2016.

**Survey year**	**Moran's index**	**Statistic (*z*-value)**	***p*** **-value**
2005	−7.44 * 10^−2^	−1.090	0.2755
2011	8.1 *10 ^−3^	0.478	0.3699
2016	−1.1 * 10^−2^	0.0471	0.9431

Table 4 depicted that the neighboring zones are independent (*p*-value > 0.05) for the three consecutive survey years.

### Ordinal logistic regression

There are different types of an ordinal logistic regression models. These are the proportional odds model (POM), partial proportional odds model (PPOM), adjacent category logit model (ACLM), and generalized ordered logit model (GOLM). Thus, before estimating parameters using the appropriate statistical approach, the model comparison was carried out using AIC and LRT (see Table 5).

**Table 5:** Model comparison.

**Type of model**	**Observation**	**AIC values**	**LRT**	***p*** **-value**
POM	8,421	14,033.633	1,140.796	0.001
PPOM	8,421	13,877.24	1,345.001	0.001
ACLM	8,421	14,016.853	1,157.576	0.001
GOLM	8,421	13,898.918	1,316.429	0.001

All models were significant in the final fit relative to their intercept and covariate models, as indicated by a significant LR test. A model with the smallest value of AIC or with the largest values of LRT was considered a good model and preferable. PPOM has the lowest AIC and highest LR values (13,877.24 and 1,345.001, respectively) compared to the others (Table 5). For ordinal dependent variables with J categories, there are J−1 binary models to conduct a series of comparisons. In this study, the response variable has three categories and there are two possible binary comparisons: moderate anemia and above vs. mild anemia and non-anemic and moderate and mild anemia vs. non-anemic.

### Partial proportional models

The logistic command was used to fit the partial proportional model. In this model, restrictions of parallel lines were imposed on some variables to meet the assumption while others, like wealth and EDHS, were not. The PPOM used a series of Wald tests to check the assumption of proportionality for the categories of all explanatory variables (see Tables 6, 7).

**Table 6:** Maximum likelihood estimates of partial proportional odds model.

**Predictors**	**Category**	**Coefficient**	***p*** **-value**	**OR**	**95% CI (OR)**
**Moderate and above vs. mild and not anemic**					
Wealth	Poorest (ref.)	0.0000		1.000	
	Poorer	−0.0233	0.7425	0.977	0.850–1.122
	Middle	−0.17320	0.0292^*^	0.841	0.720–0.983
	Richer	−0.1255	0.1103	0.882	0.756–1.029
	Richest	−0.7140	< 0.0001^*^	0.490	0.409–0.586
Religion	Muslim (ref.)	0.0000		1.000	
	Catholic	−0.2591	0.6021	0.772	0.291–2.044
	Orthodox	0.0456	0.7258	1.047	0.811–1.351
	Other	1.3793	0.0967	3.972	0.7802–0.221
	Protestant	0.0111	0.9357	1.011	0.772–1.324
	Traditional	−0.1031	0.7243	0.902	0.509–1.600
EDHS	2005	−0.0251	0.709	0.975	0.855–1.113
	2011	−0.0356	<0.001^*^	0.701	0.631–0.777
	2016 (ref.)	0.0000		1.000	
Region	SNNP	−0.1314	0.6246	0.877	0.518–1.484
	Addis Ababa	−0.8263	0.1795	0.438	0.131–1.463
	Afar	1.2686	0.0005^*^	3.556	1.735–7.286
	Tigray	−0.2688	0.4386	0.764	0.387–1.509
	Ben-Gumz	0.3499	0.1322	1.419	0.900–2.238
	Gambella	0.5922	0.0419	1.808	1.022–3.198
	Harari	0.8888	0.0006^*^	2.432	1.467–4.033
	Oromiya	−0.1309	0.5766	0.877	0.554–1.389
	Somalia	1.0097	0.0017^*^	2.745	1.460–5.158
	Dire-Dawa	1.6990	< 0.0001^*^	5.469	3.307–9.043
	Amhara (ref.)	0.0000		1.000	
Household member	1–5 (ref.)	0.0000		1.000	
	4–6	−0.0990	0.5076	0.906	0.676–1.214
	7–9	0.0704	0.5234	1.073	0.864–1.332
	≥10	−0.0513	0.6418	0.950	0.765–1.179
SDW	Unimproved	0.1758	0.0128^*^	1.192	1.038–1.369
	Improved (ref.)	0.0000		1.000
Age group	<20 (ref.)	0.0000		1.000
	20–29	−0.6335	0.0072^*^	0.531	0.334–0.842
	30–39	−0.5598	0.0179^*^	0.571	0.359–0.908
	40–49	−0.6133	0.0155^*^	0.542	0.33–0.89
	Constant	−1.8966	< 0.0001^*^	0.150	0.082–0.276

* Significant variable.

SDW, Source of Drinking Water; EDHS, Ethiopian Demographic and Health survey year; ref., Reference.

**Table 7:** Maximum likelihood estimates of partial proportional odds model.

**Predictors**	**Category**	**Coefficient**	***p*** **-value**	**OR**	**95% CI OR**
**Mild and above vs. not-anemic**					
Wealth	Poorest (ref.)	0.0000		1.000
	Poorer	−0.0233	0.7425	0.9770	0.850–1.122
	Middle	−0.1732	0.0292^*^	0.841	0.720–0.983
	Richer	−0.1255	0.1103	0.882	0.756–1.029
	Richest	−0.7140	< 0.0001^*^	0.490	0.409–0.586
Religion	Muslim (ref.)	0.0000		1.000
	Catholic	−0.9536	0.0123^*^	0.385	0.183–0.813
	Orthodox	−0.0146	0.8762	0.986	0.820–1.184
	Other	−2.1768	0.0015^*^	0.113	0.029–0.436
	Protestant	−0.0455	0.6501	0.956	0.785–1.163
	Traditional	−0.0967	0.6625	0.908	0.588–1.401
EDHS	2005	−0.0251	0.709	0.975	0.855–1.113
	2011	−0.0356	< 0.001^*^	0.701	0.631–0.777
	2016 (ref.)	0.0000		1.000	
Region	Amhara (ref.)	0.0000		1.000
	SNNP	−0.1092	0.5735	0.897	0.613–1.311
	Addis Ababa	−0.6401	0.0702	0.527	0.264–1.054
	Afar	0.7497	0.0170^*^	2.116	1.143–3.918
	Tigray	1.2617	< 0.0001^*^	3.531	2.393–5.211
	Ben-Gumz	0.1087	0.5249	1.115	0.797–1.559
	Gambella	0.5685	0.0075^*^	1.766	1.164–2.679
	Harari	0.7188	0.0002^*^	2.052	1.404–3.000
	Oromiya	−0.2396	0.1588	0.787	0.564–1.098
	Somalia	0.2953	0.2744	1.344	0.791–2.282
	Dire-Dawa	0.3590	0.1851	1.432	0.842–2.435
Number of household member	1–5 (ref.)	0.0000		1.000
	4–6	0.4122	0.0013^*^	1.510	1.175–1.940
	7–9	0.3592	0.0002^*^	1.432	1.183–1.734
	≥10	0.0753	0.4015	1.078	0.891–1.305
SDW	Unimproved	−0.0382	0.5515	0.962	0.849–1.092
	Improved (ref.)	0.0000		1.000
Age group	<20 (ref.)	0.0000		1.000	
	20–29	−0.1137	0.6101	0.892	0.576–1.382
	30–39	0.0968	0.6663	1.102	0.709–1.711
	40–49	0.0733	0.7560	1.076	0.678–1.708
	Constant	−1.4175	< 0.0001^*^	0.242	0.142–0.414

* Significant variable.

SDW, Source of Drinking Water; EDHS, Ethiopian Demographic and Health survey year.

The result of this study revealed that the age of pregnant women aged 20–29, 30–39, and 40–49 was 46.9% (OR = 0.531, CI: 0.334–0.842), 42.9% (OR = 0.571, CI: 0.359–0.908), and 45.8% (OR = 0.542, CI: 0.33–0.89) less likely to be moderate anemic rather than normal and mild anemic pregnant women as compared to pregnant women whose age was <20 years, respectively.

For pregnant women who lived in Afar, Somalia, Harari, and Dire Dawa region the odds of being moderate and above anemic increased by a factor of 3.556 (OR = 3.556, CI: 1.735–7.286), 2.745 (OR = 2.745, CI: 1.465.158), 2.433 (OR = 2.433, CI: 1.467–4.033), and 5.469 (OR = 5.469, CI: 3.407–9.043) times rather than not anemic and mild anemic pregnant women compared to who lived in Amhara region keep all other variables constant. Compared to pregnant women from the household who had consumed water from an improved source, the odds of being moderate and above anemia increased by a factor of 1.192 times percent (OR = 1.192, CI: 1.038–1.369) rather than mild and normal anemia among pregnant women from households who had consumed unimproved source of water holding all other variables constant. Pregnant women from Afar, Tigray, Gambella, and Harari regions were 2.116 (OR = 2.116, CI: 1.143–3.918), 3.53 (OR = 3.53, CI: 2.393–5.211), 11.766 (OR = 11.766, CI: 1.164–2.679), and 2.052 (OR = 2.052, CI: 1.404–3.00), respectively times more likely mild and above anemia rather than non-anemic pregnant women compared to pregnant women from Amhara holding all other variable constant. Compared pregnant women from 1 to 3 number household members, the pregnant women from 4 to 6 and 7 to 9 number household members were 51% (OR = 1.51, CI: 1.175–1.94) and 43.2% (OR = 1.432, CI: 1.183–1.734) more likely to be mild and above anemia, holding all other variables constant. The pregnant women whose religions were catholic and other 61.5% (OR = 0.385, CI: 0.183–0.813) and 88.7% (OR = 0.113, CI: 0.029–0.436) were less likely to be mild and above anemic rather than not anemic compared to pregnant women whose religion was Muslim keep all other variables were constant.

### Predictors that satisfied parallel line assumption

The result of PPOM revealed that holding all other variables constant, compared to the pregnant women from households with the poorest wealth index, the risk of anemia decreased by 15.9% (OR = 0.841, CI: 0.72–0.983) and 51% (OR = 0.49, CI: 0.409–0.586) for the pregnant women whose household wealth index was middle and richest respectively rather than normal. As compared to the pregnant women from the 2016 survey year, the pregnant women from the 2011 survey year were 29.9% (OR = 0.701, CI: 0.631–0.777) less likely to be moderate, severe, and mild anemic rather than not anemic, keeping all other variables constant.

## Discussion

The main purpose of this study was to investigate the associated factors and the spatial distribution of anemia among Ethiopian administrative zones using 2005, 2011, and 2016 EDHS. The factors/variables in this study were education level, age group of mother, place of residence, region, religion, source of drinking water, wealth index, cooking fuel, types of toilet, preceding birth interval, body mass index, pregnant currently working, survey years, and number of household members. The prevalence of anemia levels for pregnant women varied among regions. The highest proportion of moderate anemia and above was at Dire Dawa, followed by Afar, and mild anemia at Harari, followed by Afar. Pregnant women from Amhara, followed by Addis Ababa, had the highest proportions of non-anemia. The lowest proportions of moderate anemia and above were observed in Addis Ababa, followed by SNNP and Amhara.

In this study, the adjacent category logit model, generalized ordered logit model, partial proportional odds model, and proportional odds model were fitted to the data, and comparisons of models were made. Thus, the best fit according to AIC and LRT is PPOM, and it was used to identify significant determinants of anemia levels. Parameter estimates of the PPOM are presented and interpreted for the significant predictors (at a 5% significance level). Significant factors associated with anemia level in pregnant women include the mother's age group, region, religion, and source of drinking water; wealth index; the number of household members, and EDHS. The regional differences of distribution of maternal anemia may be because of the health facilities access, weather condition, and types of consumption across region are different.

Our study showed that the wealth index has a significant association with anemia levels among pregnant women. The study found that pregnant women from the richest and middle-class households had a lower risk of anemia than pregnant women from the poorest households. This result is in line with studies done in Ethiopia (35, 36). Based on spatial analysis, the highest prevalence of anemia was in North West Tigray, Waghimra, Oromia special woreda, West shewa, East shewa, North shewaR4, East harargie, Selti, Alaba, Sidama, Segen people, and South Omo, Afar zone5 and zone3 and Somali Siti, whereas the lowest prevalent zones were Afarzone4, Huru guduru, West harargie, Gurague, Yem, konta, KT, and Gamo Gofa but Shewa (R3) administrative zone was a high-risk area, and Sheka, Majang, Illubabor, and West Wollega were low-risk areas of anemia based on the nearest EDHS data.

The finding of this study revealed that the age of the mother had a significant effect on the anemia level of pregnant women. Pregnant women whose age was <20 years more likely to be moderate and above anemia as compared to pregnant women whose age was >20 years which is similar to a previous study conducted by Woldegebriel et al. (14). As a result of this research, a number of household members had significantly affected anemia levels among pregnant women. This indicates that as the number of household members increased, the risk of anemia among pregnant women also increased. This finding fitted with the study done in Tanzania (36).

The findings of this study revealed that the source of drinking water had a significant effect on the level of anemia among pregnant women. This showed that pregnant women who drank unimproved water were more anemic compared to those who drank improved water, which is similar to a previous study conducted by Berhe et al. (37).

The current study identified that religion was a significant effect on anemia among pregnant women. The risk of anemia among pregnant women whose religion was catholic was high compared to those whose religion was Muslim this result was consistent with the result obtained by Woldegebriel et al. (14).

This study incorporated data from three successive surveys and considered a simultaneous spatial variation of anemia on pregnant women. Thus, the findings generated from this research would improve the findings of cross-esctional studies so far and will help policymakers implement appropriate policy measures. This study has a number of drawbacks. The survey from which the data for this study were gathered was conducted in three waves of 5 years each: in 2005, 2011, and 2016. As a result, prevalence of anemia on pregnant women are noticeable within 5 years. Another weakness of the study might be attributed to the memory bias in the cross-sectional DHS data. Using the most recent survey data, we advise additional research.

## Conclusion

About over one-third of the expectant mothers (34.5%) were anemic to varying degrees. The prevalence of anemia in 2005 in the southern region of Ethiopia would be significantly impacted by anemia, according to the outcome of forecasted values. According to data from the fourth EDHS, the areas with the highest prevalence of anemia were West Shewa, Waghimra, Oromia special woreda, and North West Tigray. Anemia levels in pregnant women were significantly influenced by factors including location, wealth index, drinking water source, household size, mother's age, religion, and EDHS. Women who were expecting were less likely to develop moderate or higher levels of anemia if their income index was higher and they drank superior water. Pregnant women with 1–3 family members who were less likely to have mild or above-average anemia.

## Data availability statement

The datasets presented in this study can be found in online repositories. The names of the repository/repositories and accession number(s) can be found at: https://dhsprogram.com/data/available-datasets.cfm.

## Author contributions

MA proposed the first draft, conducted data analysis and interpretation, and wrote the manuscript. HF, DZ, and LT edited and revised the manuscript. All authors read and approved the final manuscript.

## References

[1] Lebso M Anato A Loha E. Prevalence of anemia and associated factors among pregnant women in Southern Ethiopia: A community based cross-sectional study. PLoS ONE. (2017) 12:e0188783. 10.1371/journal.pone.0188783 29228009 PMC5724831

[2] WHO. Haemoglobin Concentrations for the Diagnosis of Anaemia and Assessment of Severity. Geneva: World Health Organization (2011).

[3] Bastida JM Girós ML Benito R Janusz K Hernández-Rivas JM González-Porras JR. Sitosterolemia: Diagnosis, metabolic and hematological abnormalities, cardiovascular disease and management. Curr Med Chem. (2019) 26:6766–75. 10.2174/0929867325666180705145900 29984642

[4] Asiyah N Rosvita V. Risk factors among pregnant women. In: 1st Paris Van Java International Seminar on Health, Economics, Social Science and Humanities (PVJ-ISHESSH 2020). Paris: Atlantis Press (2021).

[5] Derso T Abera Z Tariku AJB. Magnitude and associated factors of anemia among pregnant women in Dera District: A cross-sectional study in northwest Ethiopia. BMC Res Notes. (2017) 10:1–8. 10.1186/s13104-017-2690-x 28764745 PMC5540297

[6] Tesema GA Worku MG Tessema ZT Teshale AB Alem AZ Yeshaw Y. Prevalence and determinants of severity levels of anemia among children aged 6–59 months in sub-Saharan Africa: A multilevel ordinal logistic regression analysis. PLoS ONE. (2021) 16:e0249978. 10.1371/journal.pone.0249978 33891603 PMC8064743

[7] Deriba BS Bulto GA Bala ET. Nutritional-related predictors of anemia among pregnant women attending antenatal care in central Ethiopia: An unmatched case-control study. BioMed Res Int. (2020) 2020:8824291. 10.1155/2020/8824291 33294455 PMC7691012

[8] Kay A Leidman E Lopez V Wilkinson C Tondeur M Bilukha O. The burden of anaemia among displaced women and children in refugee settings worldwide, 2013–2016. Br Med J. (2019) 4:e001837. 10.1136/bmjgh-2019-001837 31798995 PMC6861076

[9] Natekar P Deshmukh C Limaye D Ramanathan V Pawar A. A micro review of a nutritional public health challenge: Iron deficiency anemia in India. Clin Epidemiol Glob Health. (2022) 2022:100992. 10.1016/j.cegh.2022.100992

[10] Chowdhury HA Ahmed KR Jebunessa F Akter J Hossain S Shahjahan M. Factors associated with maternal anaemia among pregnant women in Dhaka city. BMC Womens Health. (2015) 15:1–6. 10.1186/s12905-015-0234-x 26395981 PMC4580087

[11] Osman MO Nour TY Bashir HM Roble AK Nur AM Abdilahi AO. Risk factors for anemia among pregnant women attending the antenatal care unit in selected Jigjiga public health facilities, Somali region, East Ethiopia 2019: Unmatched case–control study. J Multidiscip Healthc. (2020) 13:769. 10.2147/JMDH.S260398 32848406 PMC7428401

[12] Asresie MB Fekadu GA Dagnew GW. Determinants of anemia among children aged 6–59 months in Ethiopia: Further analysis of the 2016 Ethiopian demographic health survey. Adv Public Health. (2020) 2020:3634591. 10.1155/2020/3634591

[13] Agresti A. Categorical Data Analysis. New York, NY: John Wiley & Sons (2003).

[14] Woldegebriel AG Gebrehiwot GG Desta AA Ajemu KF Berhe AA Woldearegay TW. Determinants of anemia in pregnancy: Findings from the Ethiopian health and demographic survey. Anemia. (2020) 2020:2902498. 10.1155/2020/2902498 32566286 PMC7293722

[15] Getahun W Belachew T Wolide ADJ. Burden and associated factors of anemia among pregnant women attending antenatal care in southern Ethiopia: Cross sectional study. BMC Res Note. (2017) 10:1–7. 10.1186/s13104-017-2605-x 28705235 PMC5512984

[16] Dersseh WM. Agronomic and Socioeconomic Sustainability of Farming Systems: A Case in Chencha, South Ethiopia. Wageningen: Wageningen University and Research (2017).

[17] Qureshi MH. Journey in the realm of geography. In: Reflections on 21st Century Human Habitats in India. Berlin: Springer. (2021). p. 27–53. 10.1007/978-981-16-3100-9_2

[18] Liyew AM Kebede SA Agegnehu CD Teshale AB Alem AZ Yeshaw Y. Spatiotemporal patterns of anemia among lactating mothers in Ethiopia using data from Ethiopian Demographic and Health Surveys (2005, 2011 and 2016). PLoS ONE. (2020) 15:e0237147. 10.1371/journal.pone.0237147 32760116 PMC7410320

[19] Hosmer DW Lemeshow SJNY. Applied Logistic Regression. New York, NY: John Wiley & Sons (2000).

[20] Tesfaw LM Essey KM. Wealth index and other behavioral and sociodemographic characteristics associated with body mass index in Ethiopia. SAGE Open Med. (2021) 9:20503121211016156. 10.1177/20503121211016156 34094557 PMC8142017

[21] Agresti A. An Introduction to Categorical Data Analysis. New York, NY: John Wiley & Sons (2018).

[22] Agresti A. Analysis of Ordinal Categorical Data. Vol. 656. New York, NY: John Wiley & Sons (2010).

[23] Elamir E Sadeq H. Ordinal regression to analyze employees'attitudes towards the application of total quality management. J Appl Quant Methods. (2010) 5.

[24] Ananth CV Kleinbaum DG. Regression models for ordinal responses: A review of methods and applications. Int J Epidemiol. (1997) 26:1323–33. 10.1093/ije/26.6.1323 9447413

[25] Liu X. Applied Ordinal Logistic Regression Using Stata: From Single-Level to Multilevel Modeling. Thousand Oaks, CA: Sage Publications (2015).

[26] Getis A. Spatial autocorrelation. In: Handbook of Applied Spatial Analysis. Berlin: Springer (2010). p. 255–78. 10.1007/978-3-642-03647-7_14

[27] Bivand RS Pebesma E Gomez-Rubio V. Applied Spatial Data Analysis With R. Vol. 747248717. Berlin: Springer (2008).

[28] Tobler WR. A computer movie simulating urban growth in the Detroit region. Econ. Geogr. (1970) 46(Sup.1):234–40. 10.2307/143141

[29] Bivand RS Wong DWJT. Comparing implementations of global and local indicators of spatial association. Ideas. (2018) 27:716–48. 10.1007/s11749-018-0599-x

[30] Overmars KD De Koning G Veldkamp AJE. Spatial autocorrelation in multi-scale land use models. Ecol Model. (2003) 164:257–70. 10.1016/S0304-3800(03)00070-X

[31] Młodak AJJoC. K-means, ward and probabilistic distance-based clustering methods with contiguity constraint. J Classificat. (2021) 38:313–52. 10.1007/s00357-020-09370-5

[32] Carrijo TB da Silva ARJGA. Modified Moran's I for small samples. Geograph Anal. (2017) 49:451–67. 10.1111/gean.12130

[33] Wu T Li YJAG. Spatial interpolation of temperature in the United States using residual kriging. Appl Geogr. (2013) 44:112–20. 10.1016/j.apgeog.2013.07.012

[34] Aidi MN Purwaningsih TJ. Modelling spatial ordinal logistic regression and the principal component to predict poverty status of districts in Java Island. Sci Acad Publ. (2013) 3:1–8. 10.5923/j.statistics.20130301.0122499009

[35] Liyew AM Teshale ABJBPH. Individual and community level factors associated with anemia among lactating mothers in Ethiopia using data from Ethiopian demographic and health survey, 2016; A multilevel analysis. BMC Public Health. (2020) 20:1–11. 10.1186/s12889-020-08934-9 32448212 PMC7247135

[36] Sunguya BF Ge Y Mlunde L Mpembeni R Leyna G Huang J. High burden of anemia among pregnant women in Tanzania: A call to address its determinants. Nutr J. (2021) 20:1–11. 10.1186/s12937-021-00726-0 34238307 PMC8268339

[37] Berhe B Mardu F Legese H Gebrewahd A Gebremariam G Tesfay K. Prevalence of anemia and associated factors among pregnant women in Adigrat General Hospital, Tigrai, northern Ethiopia. BMC Res Notes. (2018) (2019) 12:1–6. 10.1186/s13104-019-4347-4 31151463 PMC6544916

